# Production of Hydrophobic Zein-Based Films Bioinspired by The Lotus Leaf Surface: Characterization and Bioactive Properties

**DOI:** 10.3390/microorganisms7080267

**Published:** 2019-08-16

**Authors:** Ângelo Luís, Fernanda Domingues, Ana Ramos

**Affiliations:** 1 Centro de Investigação em Ciências da Saúde (CICS-UBI), Universidade da Beira Interior, Avenida Infante D. Henrique, 6200–506 Covilhã, Portugal; 2 Laboratório de Fármaco-Toxicologia, UBIMedical, Universidade da Beira Interior, Estrada Municipal 506, 6200–284 Covilhã, Portugal; 3 Departamento de Química, Faculdade de Ciências, Universidade da Beira Interior, Rua Marquês d’Ávila e Bolama, 6201–001 Covilhã, Portugal; 4 Materiais Fibrosos e Tecnologias Ambientais (FibEnTech), Universidade da Beira Interior, Rua Marquês d’Ávila e Bolama, 6201–001 Covilhã, Portugal

**Keywords:** lotus-effect, water contact angle, food packaging, licorice essential oil, antioxidant properties, antibacterial activity, foodborne pathogens

## Abstract

Hydrophobic zein-based functional films incorporating licorice essential oil were successfully developed as new alternative materials for food packaging. The lotus-leaf negative template was obtained using polydimethylsiloxane (PDMS). The complex surface patterns of the lotus leaves were transferred onto the surface of the zein-based films with high fidelity (positive replica), which validates the proposed proof-of-concept. The films were prepared by casting method and fully characterized by Scanning Electron Microscopy (SEM), Fourier-transform infrared spectroscopy (FTIR) and Differential Scanning Calorimetry (DSC). The grammage, thickness, contact angle, mechanical, optical and barrier properties of the films were measured, together with the evaluation of their biodegradability, antioxidant and antibacterial activities against common foodborne pathogens (*Enterococcus faecalis* and *Listeria monocytogenes*). The zein-based films with the incorporation of licorice essential oil presented the typical rugosities of the lotus leaf making the surfaces very hydrophobic (water contact angle of 112.50°). In addition to having antioxidant and antibacterial properties, the films also shown to be biodegradable, making them a strong alternative to the traditional plastics used in food packaging.

## 1. Introduction

Food packaging is designed to protect food from external factors, such as temperature, light or humidity that can lead to degradation [1]. Moreover, packages also protect its content from other environmental influences, namely, odors, microorganisms, shocks, dust, vibrations and compressive forces [1].

The production and application of synthetic materials in food packaging has grown quickly over the past few decades, resulting in serious environmental concerns due to the resistance to degradation of these synthetic materials [2,3]. In recent years, the replacement of synthetic plastics by natural polymers in packaging materials has been an intense research field [3,4]. Particularly, bio-based natural polymers (mainly polysaccharides and proteins) have been given increasing attention to be used in food packaging films because of their abundance, biodegradability, biocompatibility, and non-toxicity [3,4]. However, natural polysaccharides have their weak points in making hydrophobic films because most of them are hydrophilic and water absorbing, resulting in rapid solubilization in aqueous environments [5].

Protein-based films have several advantages over other types of edible materials. Proteins are constructed of nearly 20 different amino acids, and usually have good film forming capability [6]. Moreover, protein-based films are generally good gas barriers, possess good mechanical properties, and can also be regarded as nutrients [6].

Zein, a by-product obtained from corn starch processing, is prepared from corn protein flour. Zein is particularly rich in hydrophobic and neutral amino acids as well as some sulfur-containing amino acids, but lacks polar or ionizable amino acids [7]. Due to its large number of hydrophobic groups, zein is soluble in aqueous ethanol, yet insoluble in pure water. Since it possesses well-known film-forming ability caused by its unique amino acid composition, it is widely used in food packaging materials [7]. Pure zein films have good water barrier properties, but their mechanical properties are relatively poor. In order to overcome these deficiencies, blend zein films with other biodegradable biopolymers has been widely studied, and the zein provided good potential to produce blend films [7].

Superhydrophobic surfaces are used by several living organisms, both animals and plants. The most famous case from the plant kingdom (*Plantae*) is the lotus leaf (*Nelumbo nucifera*) [8]. The extreme water repellency of lotus leaves stems from the combination of low surface energy with the hierarchical topology present on the leaf surface [9]. Water droplets roll freely on these surfaces and remove dirt, keeping the leaves clean, even in the muddy waters where these plants tend to grow [9]. This extreme water repellency and self-cleaning performance of the lotus leaf is usually known as the “lotus effect” [10]. Recently, researchers have focused on recreating these surfaces, structured at the micro and nanometric scale, with new functionalities, replicating or mimicking the hierarchical surface morphology of the lotus leaf [11].

Licorice (*Glycyrrhiza glabra* L.) is a plant belonging to the *Fabaceae* family. Its sweet flavor makes it a popular ingredient in the production of candies and sweets in Europe [12]. The antioxidant and antibacterial properties of the licorice essential oil (EO) have already been demonstrated, together with its incorporation on carboxymethyl xylan films with potential to be used as novel food packaging materials [13].

Therefore, the aim of this work was to develop hydrophobic zein-based films incorporating licorice EO while biomimicking the lotus leaf surface, which is the innovation of this work. The films obtained were then characterized, and their bioactive properties evaluated.

## 2. Materials and Methods 

### 2.1. Reagents

Polydimethylsiloxane (PDMS)—Sylgard^®^ 184 Silicone Elastomer was obtained from Dow Corning (Midland, MI, USA). Zein from maize (CAS Number: 9010-66-6), presenting a molecular weight of 22–24 kDa, was supplied by Sigma-Aldrich (Saint Louis, MO, USA). Glycerol (anhydrous extra pure) was purchased from Merck (Darmstadt, Germany). The licorice EO (*Glycyrrhiza glabra* L.) (*Leguminosae*/*Fabaceae*) was obtained from Best Formula Industries (BF1, Kuala Lumpur, Malaysia). The EO (Pure Essential) was isolated from trunks of the plant by steam distillation and was stored at −20 °C in the dark until analysis and further use. The purity of the EO was tested and its quality ensured to be consistent with the standards described in the European Pharmacopoeia, being suitable to be used in products for human consumption. The chemical composition, analyzed by Gas Chromatography coupled to Mass Spectrometry (GC-MS, Perkin Elmer Clarus 600, Shelton, CT, USA), and the bioactivities, namely antioxidant ant antibacterial, of this EO were previously published [13].

### 2.2. Lotus Leaves

The fresh lotus leaves (*Nelumbo nucifera*) were collected in Autumn 2018 (September-October) and were kindly provided by the Jardim Botânico of Universidade de Coimbra. The hierarchical surface morphology of the lotus leaf was observed by Scanning Electron Microscopy (SEM, Hitachi, Chiyoda, Japan). For that, pieces of lotus leaves were fixed with 5% (*v*/*v*) glutaraldehyde at 4 °C for 12 h. After that, samples were washed once with phosphate buffer saline (PBS) and the dehydration was carried out in ethanol series for 1 h each (30, 50, 70, 80, 90% (*v*/*v*), and absolute) at room temperature [14,15]. The samples were then allowed to dry overnight in a desiccator. Finally, samples were coated with gold and analyzed by VP SEM Hitachi S-3400N (Hitachi, Chiyoda, Japan) using a voltage of 20.0 kV and 100.0 μA emission.

### 2.3. Negative Template Fabrication

**Figure 1:**
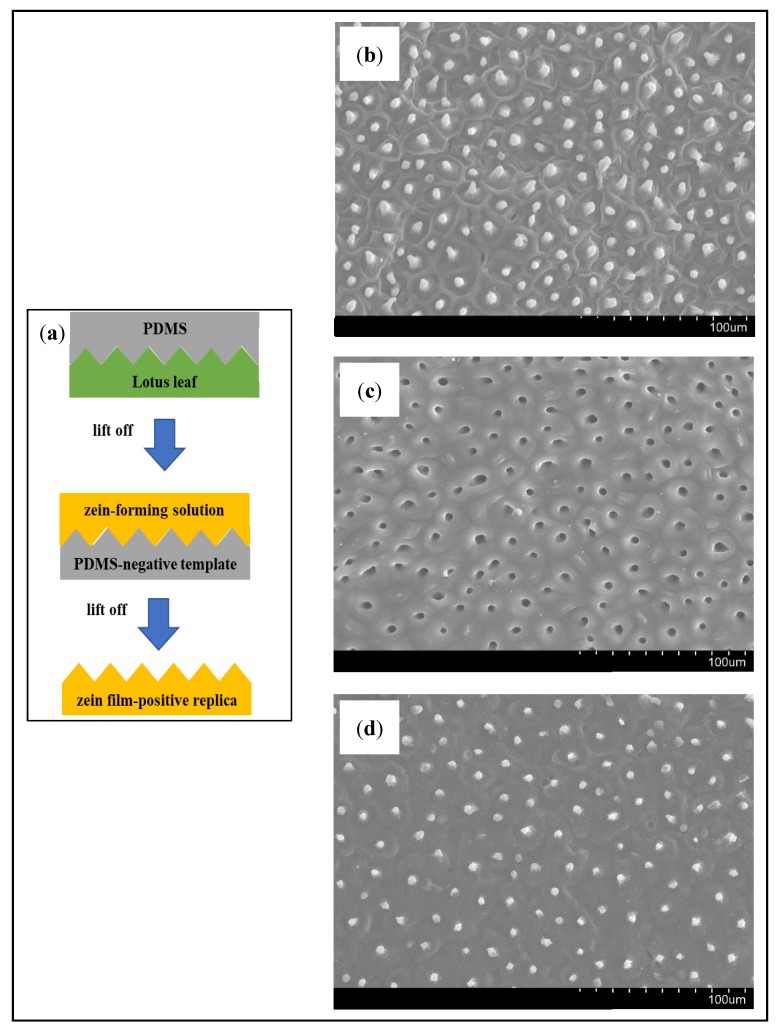
Flowchart for negative template fabrication and films preparation (**a**). SEM images of lotus leaf surface (**b**), PDMS-negative template (**c**), and zein film-positive replica (**d**). (Magnification: 500×).

PDMS was used to produce the lotus leaf negative template. The lotus leaves were gently cut and glued to glass Petri dishes. Then, molten paraffin was placed on the edges of the Petri dishes to seal the samples and prevent the polymer from dripping. Initially, the mixture of PDMS and its catalyzer (10:1; *w*/*w*) were weighed and mixed by hand for 5 min in order to improve homogeneity. Then, this mixture was degassed under vacuum until visible air bubbles formed during the mixing procedure had disappeared (30 min) [16]. Finally, the mixture was cast on the previously prepared Petri dishes with the lotus leaves [17]. The Petri dishes were then placed into a ventilated oven for a curing time of 4 h at 60 °C and then were left for more 48 h at room temperature [16] (Figure 1a). The negative template produced was visualized by SEM. Small pieces of the negative template were directly mounted on stubs and then coated with gold and analyzed by VP SEM Hitachi S–3400N using a voltage of 20.0 kV and 100.0 μA emission. 

### 2.4. Preparation of Zein-Based Films 

The film-forming solution was prepared by dissolving zein powder in 80% (*v*/*v*) ethanol-water solution (10%; *w*/*v*) under magnetic stirring for 30 min at 80 °C and 250 rpm. Glycerol at 25% (g glycerol/g dry zein powder) was added to the solution as plasticizer agent and stirred for 8 min at 80 °C and 250 rpm [18]. Then, licorice EO at 30% (g EO/g dry zein powder) was incorporated and was stirred again (8 min, 80 °C, 250 rpm) [13,18]. Films were then obtained by casting. For this purpose, the film-forming solution was spread on polystyrene Petri dishes in which the lotus leaf negative template was mounted as described above (Figure 1a), allowing the obtention of zein-based films with similar hierarchical surface morphology to that of the lotus leaf (positive replica). Films were also obtained by spreading the film-forming solution on simple polystyrene Petri dishes. Finally, the Petri dishes were placed into a ventilated oven to dry the mixture (24 h, 40 °C). The positive replica films obtained were observed by SEM as described for the negative template.

### 2.5. Characterization of Films

The control film (without EO) and the film with EO, both casted on simple polystyrene Petri dishes and on lotus negative template, were further characterized.

#### 2.5.1. Fourier-Transform Infrared Spectroscopy (FTIR)

FTIR spectra of the films were obtained between 4000 and 600 cm^−1^ using a Nicolet iS10 smart iTRBasic (Thermo Fisher Scientific, Waltham, MA, USA) model, with 64 scans and a 4 cm^−1^ resolution [19].

#### 2.5.2. Differential Scanning Calorimetry (DSC)

DSC analysis of the films was performed on a calorimeter (Netzsch DSC 204, Selb, Germany) operating in the following conditions: heating rate of 2 °C/min, inert atmosphere, and temperature range from 22 to 350 °C. For all the analyses, the respective baselines were firstly obtained [20]. 

#### 2.5.3. Grammage, Thickness and Mechanical Properties

The grammage of the films was calculated based on the ratio between their mass and area (g/m^2^), according to ISO 536:1995. The thickness (µm) was measured according to ISO 534:2011 using a micrometer (Adamel Lhomargy Model MI 20, Veenendaal, Netherlands), with several random measurements being considered. Tensile strength (MPa), elongation (%) and elastic modulus (MPa) of the films were obtained using a tensile tester (Thwing-Albert Instrument Co., West Berlin, NJ, USA), at 23 ± 2 °C and 50 ± 5% relative humidity (RH), as per ISO 1924/1 with a single modification. The initial grip was set at 50 mm and the crosshead was set at 10 mm/min [21].

#### 2.5.4. Optical Properties

The color and transparency of the films were evaluated using a Technidyne Color Touch 2 spectrophotometer (New Albany, IN, USA). Measurements were performed on at least three random positions of the films using the D65 illuminant and 10° observer. Color coordinates L* (lightness), a* (redness; ±red-green) and b* (yellowness; ±yellow-blue) were obtained. The transparency of the films was calculated according to the equation defined in ISO 2289 [21].

#### 2.5.5. Barrier Properties: Water Vapor Permeability 

Water vapor permeability (WVP; g/(Pa.day.m)) and water vapor transmission rate (WVTR; g/(m^2^.day)) were determined according to the standard protocol ASTM E96-00. The films, which had been equilibrated at 23 ± 2 °C and 50 ± 5% RH for 72 h, were fixed on the top of equilibrated cups containing a desiccant (15 g of anhydrous CaCl_2_, dried at 105 °C for 2 h before being used). The test cups were placed in a cabinet at 23 ± 2 °C and 50 ± 5% RH. The weight changes were monitored for every 2 h over 48 h. The gradient was calculated from the slope of a linear regression of the weight increase versus time [22,23]. WVTR and WVP were calculated according to the following equations: 

WVTR = (∆m/∆t)/A, where Δm is the weight changes of test cups (g), A is the test area (m^2^) and t is test time (day).

WVP = WVTR/∆*p* = (WVTR/[*p*(RH_1_ − RH_2_)]) × e, where *p* is the vapor pressure of water at 23 °C (Pa), RH_1_ is the RH of the cabinet (50%), RH_2_ is the RH inside the cups (0%) and e is the thickness (m) of the films.

#### 2.5.6. Contact Angle Measurement

Contact angle measurements were performed using the sessile drop method with distilled water as test liquid (OCAH 200, DataPhysics, Filderstadt, Germany). At least eight measurements were made for each film [21]. Moreover, the contact angle was measured for both sides (upper and lower) of each film. 

#### 2.5.7. Water Solubility

The films solubility in water was defined by the content of dry matter solubilized after 24 h of immersion in water. The initial dry matter content of each film was determined by drying to constant weight in an oven at 105 °C. Two disks of films (2 cm of diameter) were cut, weighed and immersed in 50 mL of water. After 24 h of immersion at 20 °C with occasional magnetic stirring, the pieces of films were taken out (by filtration) and dried to constant weight in an oven at 105 °C, in order to determine the weight of dry matter which was not solubilized in water [24]. The measurement of solubility of the films was determined as follows:

S(%) = [(mi − mf)/(mi)] × 100%, where S is the solubility, mi is the initial mass and mf the final mass.

#### 2.5.8. Antioxidant Activity

The antioxidant activity of the films was evaluated by the 2,2-diphenyl-1-picrylhydrazyl (DPPH) free radical scavenging assay and the β-carotene bleaching test.

For the DPPH free radical scavenging assay, 3 disks of the film (6 mm of diameter) were added to 2.9 mL of a DPPH methanolic solution (0.1 mM). Then, the absorbances were measured at 517 nm every 30 min, for 5 h, against a blank of methanol [13]. 

For the β-carotene bleaching test, 500 μL of a β-carotene solution (20 mg/mL in chloroform) were added to 40 μL of linoleic acid, 400 μL of Tween 40 and 1 mL of chloroform. The chloroform was then evaporated under vacuum, and 100 mL of oxygenated distilled water were added to the mixture to form an emulsion. Then, 5 mL of this emulsion were pipetted into test tubes containing 3 disks of the film (6 mm of diameter). Finally, the tubes were shaken and placed at 50 °C in a water bath for 1 h. The absorbances of the samples were measured at 470 nm [13].

#### 2.5.9. Antibacterial Properties

The antibacterial properties of the films against two foodborne pathogens (*Enterococcus faecalis* ATCC 29212 and *Listeria monocytogenes* LMG 16779) were evaluated by solid diffusion assay. For this test, inoculums were prepared by suspending bacteria in a sterile saline solution (NaCl; 0.85%; w/v) to a cell suspension of 0.5 McFarland (1–2 × 10^8^ colony-forming units/mL (CFU/mL)). Disks of the films (6 mm of diameter) were prepared under aseptic conditions. Then, the Müeller-Hinton agar (MHA) plates were inoculated and allowed to dry, being the disks of films placed over the inoculated culture medium. Finally, the plates were incubated at 37 °C for 24 h. After the incubation, all the plates were visually checked for inhibition zones, being their diameters measured using a pachymeter. This assay was performed three independent times [13,21].

#### 2.5.10. Biodegradability

Soil burial degradation test was carried out to evaluate the biodegradability of the films. Small pieces of films (dimensions 2 × 7 cm) were buried in natural soil at a depth of 10 cm (23 ± 2 °C and 50 ± 5% RH). After 10 days, the samples were collected, washed several times with distilled water and then allowed to dry in an oven at 50 °C for 24 h [25]. The weight loss was monitored and the FTIR spectra of the degraded films were also recorded as described above. 

### 2.6. Statistical Analysis

The results were expressed as mean ± standard deviation (SD). The data were analyzed using the statistical program IBM SPSS Statistics 25 (https://www.ibm.com/analytics/spss-statistics-software). The significant difference among means was analyzed by Student’s T-test (assuming the normal distribution of the continuous variables). A level of *p*-value < 0.05 was considered significant. Linear regression was also performed for antioxidant activity of the films measured by DPPH scavenging assay considering the % Inhibition as a function of time.

## 3. Results and Discussion

### 3.1. Proof-of-Concept: Negative Template and Positive Replica

This work intended to develop zein-based films incorporating licorice EO with potential applications in food packaging materials. Considering that these bio-based films are usually hydrophilic, and considering the “lotus effect”, it was decided to try to mimic the hierarchical surface morphology of the lotus leaves in these films.

Firstly, the surface of the leaves was analyzed by SEM (Figure 1b), where it was possible to notice the common pattern of lotus leaves with its micro and nanostructures, measuring between 7 and 8 µm. Then, to prepare a negative template of the lotus leaves, a mixture of PDMS and its catalyzer was nano-casted on fresh lotus leaves. After solidification of PDMS, the negative template was lift off and visualized by SEM (Figure 1c). The PDMS-negative template presents the holes corresponding to the structures observed in the lotus leaves surface, which means that a complementary topographic surface structure of the original template (lotus leaves) was successfully obtained. Finally, the zein-based films (with and without licorice EO) were casted on the PDMS-negative template to replicate the hierarchical surface morphology of the lotus leaves in the films. The films obtained were once again visualized by SEM (Figure 1d), ant it was possible to observe that the complex surface patterns of the lotus leaves were transferred to the surface of the zein-based films with high fidelity (positive replica), which validated the original concept behind this study. Moreover, the structures obtained in both types of zein-based films presented similar dimensions (≈7–8 µm), measured by SEM, indicating that the incorporation of the licorice EO in the films did not interfere with the nano-casting process. Other researchers had previously replicated the so-called lotus-leaf-like structures for biomedical and pharmaceutical applications [10,26,27,28].

### 3.2. FTIR and DSC Analyses of The Films

**Figure 2:**
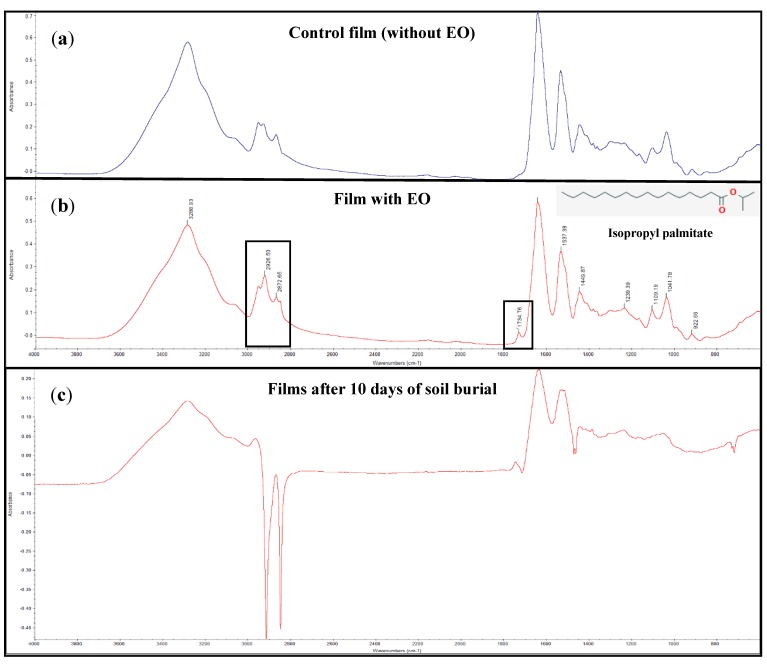
FTIR spectra of the films: control film (without EO) (**a**), film with EO (**b**), and films after 10 days of soil burial (**c**).

The spectra of zein-based films with or without licorice EO, and both casted on simple polystyrene Petri dishes and on lotus negative template were recorded. It was observed that all the films without incorporation of licorice EO presented analogous FTIR spectra. Similar results were found for the films with licorice EO, indicating that the support material where the films were casted (polystyrene or PDMS) did not influence the chemical interactions between the components of the films. In addition, both sides of each film presented similar FTIR spectra, which means that the topographic surface structure did not affect the chemical composition of the films. Figure 2a shows the FTIR spectrum of the zein film without licorice EO, while Figure 2b presents the spectrum of the film incorporating the EO. The licorice EO is mainly constituted (71.8%) by isopropyl palmitate [13], also shown in Figure 2b. The large absorption at 2960 cm^−1^ in the zein film with licorice EO was attributed to the stretching vibration of the aliphatic compounds, and the peak at 1740 cm^−1^ was ascribed to the stretching vibration of the ester compounds. Isopropyl palmitate possesses an aliphatic chain with several methyl groups (CH), while being also an ester presenting a carbonyl group (C = O) [13]. These molecular features of the main compound of the EO are responsible for the peaks visible in the FTIR spectrum of the zein-based film incorporating licorice EO, when compared to the spectrum of the control film, indicating that the EO was successfully incorporated.

**Figure 3:**
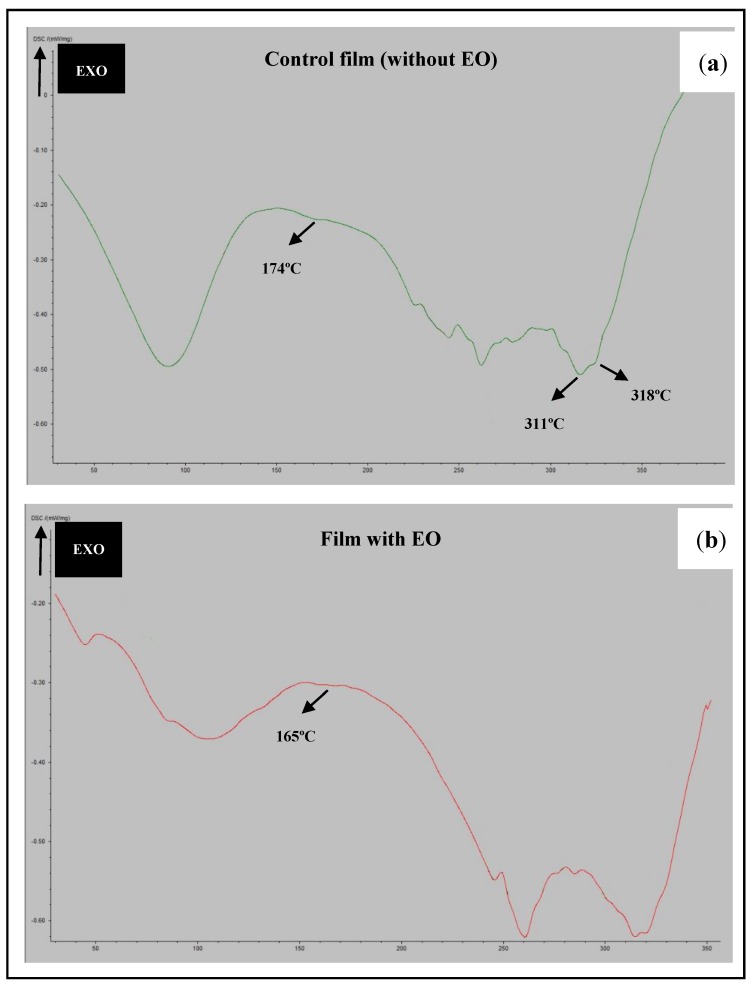
DSC curves of the films: control film (without EO) (**a**), and film with EO (**b**).

The thermal profiles of zein films with or without the licorice EO incorporated were evaluated by DSC. Similar to what was observed in FTIR analysis, the support material where the films were casted did not influence the DSC curves. Figure 3a shows the DSC curve of a zein film without EO and Figure 3b shows the DSC curve of a zein-based film incorporating licorice EO. Both thermograms show endothermic peaks in the range of temperature of 50 °C to 150 °C. The presence of such peaks is attributed to the loss of volatile components, like water, or the possibility of chain relaxation. Additionally, in this range of temperature, the breakdown of hydrogen bonds that are present in the zein structure and other molecular associations also happens [20]. Proteins have some features associated to their different tridimensional structure, such as the denaturation process. The DSC curve of zein-based film without EO (Figure 3a) exhibited endothermic peaks at 311 °C and 318 °C, which can be interpreted as the protein unfolding, similar to what was previously observed [20]. Moreover, through the DSC curve of the control film, at the 174 °C mark, a slight modification on the linear profile of the zein curve appears, which is linked to the zein glass transition temperature (Tg), since above this temperature the protein chains of zein enter in a flexible stage [20]. When incorporating licorice EO in the zein films, the DSC curve (Figure 3b) changed, having observed a lower value of Tg (165 °C), which is probably related to the loss of some flexibility in the films with the EO [20]. This fact can be explained by the interactions among zein and the compounds of licorice EO after the preparation of the films, as it was also observed by the FTIR analysis. Similar results were previously obtained by other authors dealing with zein-based films [29].

### 3.3. Grammage, Thickness, Mechanical and Optical Properties of The Films

The grammage and thickness were significantly higher (*p*-value < 0.05) in the films incorporating licorice EO casted on the lotus negative template (Table 1), which is probably due to the imprisonment of air microbubbles during the casting. The film-forming solution must fill all the holes of the lotus negative template, which are full of air that can become entrapped in the film matrix. Moreover, the incorporation of the EO in the films most likely increases their apparent density, which is also reflected in higher values of grammage.

Concerning the mechanical properties, zein films are brittle, and thus, plasticizers are needed to improve their flexibility [30]. In general, tensile strength and elongation are key factors in packaging materials, which keep their integrity during packaging, transport, storage and sales [7]. The tensile strength of the films casted on lotus negative template was significantly lower (*p* < 0.05) when compared to the films casted in simple polystyrene Petri dishes (Table 1), incorporating (or not) licorice EO. This means that it is not the licorice EO that affects tensile strength, but the support material where the films were casted, probably because of the same effect of air microbubble entrapment, as explained above.

Nevertheless, elongation and elastic modulus were similar within all the films (Table 1). Finally, the mechanical properties of the zein-based films developed in this work, using glycerol as plasticizer, are very close to the ones obtained previously for zein films using oleic acid as the plasticizer agent [29,30], but improved upon than the ones obtained using other plasticizers (buriti oil, macadamia oil and olive oil) [29].

The optical properties (L*, a* and b*) of the films with EO were only slightly affected (Table 1), since zein is a yellow powder and the licorice EO is yellow-brownish. On a previous work, the licorice EO was incorporated in carboxymethyl xylan films (white) and the optical properties were significantly changed by the EO, particularly transparency [13]. In the present work, the color of the EO is not a problem since zein is also yellow.

### 3.4. Barrier Properties: Water Vapor Permeability

The barrier properties of the films, and particularly water permeability, plays a critical role in evaluating the practical applications of functional films. It was expected that the films with licorice EO presented lower water permeability since the hydrophobic nature of the EO was known to contribute to the decrease of WVP [22]. Furthermore, in the present work, the WVP was significantly higher (*p*-value < 0.05) in the zein films casted on lotus negative template, compared to the ones casted on simple polystyrene Petri dishes (Table 2). The WVP of the films casted on polystyrene is near 2.74 × 10^−6^ g/(Pa.day.m) while for the films casted on lotus negative template it is about 3–4 × 10^−6^ g/(Pa.day.m), indicating that the water barrier properties of the films were weakened. The support material where the films were casted seems to influence the WVP, probably because of the higher values of thickness obtained for the films casted on lotus negative template. The zein-based films developed in the present work presented strong water barrier properties than the ones obtained previously with chitosan-zein edible films incorporated with anise, orange and cinnamon essential oils (250 ppm) [31].

### 3.5. Contact Angle and Water Solubility

Understanding the wettability of the films is often carried out by measuring the contact angle formed between a liquid drop and the film [32]. Additionally, the water contact angle measurements provide information about the hydrophobicity/hydrophilicity of the surface of the films. A hydrophobic surface is a surface in which the water contact angle is higher than 90° [5]. Table 3 summarizes the water contact angle measured in both sides of the films (with and without the licorice EO), casted on polystyrene and on lotus negative template. The addition of licorice essential oil increases significantly (*p*-values < 0.05) the contact angle of the films (Table 3). In films casted on simple polystyrene Petri dishes, the contact angle raises from 48.27° (without EO) to 71.80° (with EO). These results may be explained by the chemical composition of the licorice EO, mainly constituted by isopropyl palmitate, which presents an octanol/water partition coefficient (logP) of 8.16, an indicator of its strong hydrophobic character [13]. Licorice is usually taken as a hydrophobic component of edible films and coatings [33]. More interestingly was the effect observed in the films casted on lotus negative template. The upper side of the films (without the hierarchical surface morphology of the lotus leaves), presented contact angles of 65.15° (without EO) and 58.04° (with EO); contrariwise to what was verified in the lower side of the films (with the hierarchical surface morphology of the lotus leaves) that presented significantly higher (*p*-value < 0.05) contact angles than the ones obtained for the films casted on polystyrene (Table 3). The film without EO that presents the complex surface patterns of the lotus leaves (positive replica) had a contact angle of 81.95°; significantly higher (*p*-value < 0.05) contact angle was obtained for the film with EO, which is also a lotus positive replica (112.50°) (Table 3). Here, the strong hydrophobic effect of licorice/isopropyl palmitate was noticed, in addition to the “lotus effect”, which confirms the initial idea behind this work. Combining the “lotus effect” with the incorporation of the licorice EO resulted in a near superhydrophobic surface (water contact angle superior than 150°) [5]. To the best of our knowledge, it was the first time that zein-based films were obtained with such high water contact angle.

Water solubility is a measure of the resistance of the films to water [24]. The results obtained showed that all the films presented a water solubility of about 20% (Table 3), indicating the lower affinity to water of these films, which also corroborates the water contact angles obtained for the films.

### 3.6. Antioxidant and Antibacterial Properties

**Figure 4:**
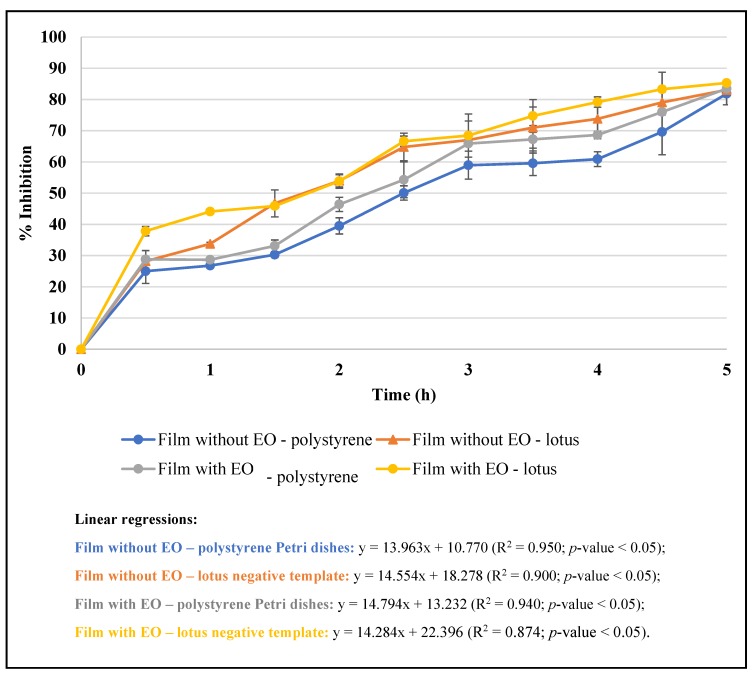
Antioxidant activity of the films measured by DPPH scavenging assay.

Figure 4 shows the antioxidant activity of the films measured by DPPH scavenging assay over time. All the films presented the ability to scavenge the free radicals in a time-dependent manner, as demonstrated by the linear regressions (*p*-values < 0.05). After 5 h of reaction, the films presented near 90% of inhibition of DPPH free radicals (Figure 4). Although the licorice EO is a potent antioxidant [13], the films without it also presented the capacity to scavenge free radicals, which is due to zein. The antioxidative nature of zein was previously reported, being frequently used in food packaging (edible films and coatings) without adding any other antioxidant during processing [34]. Some studies have reported the benefits of using zein films as packaging material for cooked turkey and fresh broccoli [34]. The capacity of the films to inhibit lipid peroxidation was also evaluated (Table 4). The results showed that the films with licorice EO have significantly higher (*p*-value < 0.05) percentages of inhibition than the control films. Both types films (polystyrene and lotus negative template) incorporating the EO presented similar capacity to inhibit the lipid peroxidation measured by the β-carotene bleaching test (20–30%), contrariwise to what was obtained with the control films (3–5%) (Table 4). This capacity can be attributed to isopropyl palmitate, the major compound of licorice EO, which is generally used in cosmetic and food industries due to its binder and fragrant properties, together with skin-conditioning and emollient activities. The Food and Drug Administration (FDA) considers that this compound is not ecotoxic and classifies it as not expected to be potentially toxic or harmful, presenting a low human health priority [35].

Also, licorice is generally recognized as safe (GRAS) by the FDA, indicating that there is no evidence, in the available information on licorice, that identifies a hazard to the public when it is used at levels that are now current and in the manner now practiced [36].

The antibacterial properties of the films were evaluated against two well-known foodborne pathogens (*E. faecalis* ATCC 29212 and *L. monocytogenes* LMG 16779). *E. faecalis* is a commensal of the human gastrointestinal tract that can persist in the external environment and is a leading cause of several infections. Given its diverse habitats, the organism has developed numerous strategies to survive a multitude of environmental conditions [37]. *L. monocytogenes* is a foodborne pathogen responsible for a disease called listeriosis, which is potentially lethal in immunocompromised people and can provoke septicemia, meningitis and fetal infection or abortion in infected pregnant women [38].

The results of the antibacterial activity studied by solid diffusion assay are presented in Table 4. Both types of zein films incorporating licorice EO presented significantly higher (*p*-value < 0.05) diameters of inhibition zones for the two bacterial species. Moreover, the anti-biofilm potential of zein films against the same foodborne pathogens was evaluated by SEM, forming the bacterial biofilms directly on the surface of the films (results not shown). It was possible to verify that in the films presenting the hierarchical surface morphology of the lotus leaves, the bacterial adhesion did not occur. More than inhibiting the bacterial growth, the films inhibited the adhesion. The anti-bio adhesion of surfaces with lotus-leaf-like rugosities is well described in the literature [39].

Since zein-based films incorporating licorice EO were able to scavenge free radicals to inhibit lipid peroxidation and the growth of foodborne pathogens, they can potentially be used as alternative food packaging systems, particularly in foods with high contents of lipids.

### 3.7. Biodegradability

**Figure 5:**
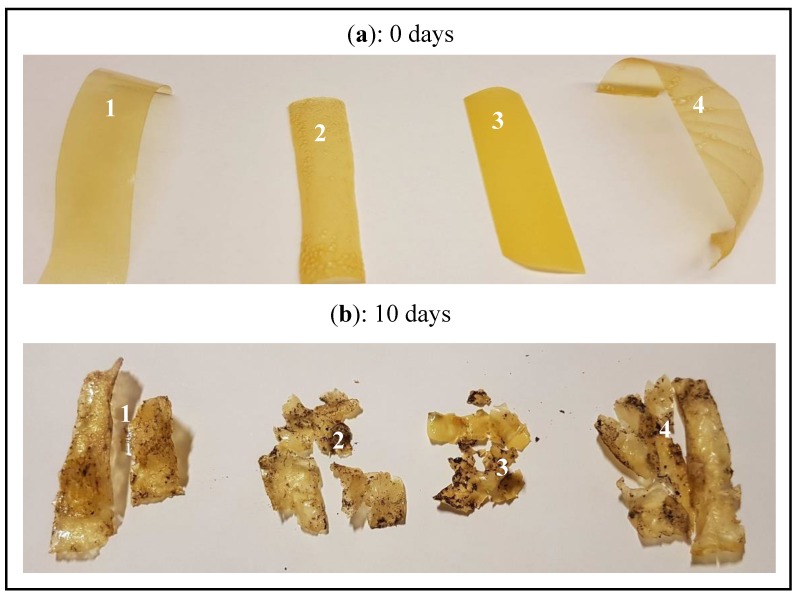
Biodegradability: soil burial degradation test of the films. Film without EO — polystyrene Petri dishes (1), film without EO — lotus negative template (2), film with EO —polystyrene Petri dishes (3), and film with EO —lotus negative template (4); initial films at 0 days (**a**), and films after soil burial degradation test (10 days) (**b**).

The biodegradability of the films was studied by soil burial degradation test for 10 days. The weight loss of zein-based films was about 50–60% (Table 3), which is a clear reflection of the biodegradation process performed by the microorganisms and moisture present in the soil. Moreover, at the end of the biodegradability test, the films were thin and appeared disrupt (Figure 5b) when compared to the initial samples (Figure 5a). The FTIR spectra of the films after the soil burial degradation test (Figure 2c) showed peaks absorption and decrease in intensities as biodegradation took place, as other authors also reported [25]. These results clearly show the biodegradable nature of the zein-based films now developed.

## 4. Conclusions

In this work, a simple and rapid method to mimic the lotus leaf surface was developed and applied to the production of zein-based films. The licorice EO was also incorporated into the films as a bioactive agent. The zein films produced using the lotus negative template presented lotus-leaf-like rugosities, resulting in very hydrophobic surfaces (water contact angle of 112.50°). The zein films with licorice essential oil are biodegradable and possess antioxidant and antibacterial properties against known foodborne pathogens, making them potential alternatives to the conventional plastics used in food packaging solutions, reducing environmental pollution and increasing the shelf-life of foods.

Future research is needed to identify ways to produce on a large scale films with the hierarchical surface morphology of the lotus leaf.

## Figures and Tables

**Table 1:** Grammage, thickness, mechanical and optical properties of the films.

Properties	Films				*p*-Values
	Polystyrene Petri Dishes		Lotus Negative Template		
	Without EO ^a^	With EO ^b^	Without EO ^c^	With EO ^d^	
Grammage (g/m^2^)	107.30 ± 5.33	145.10 ± 18.64	128.55 ± 3.35	167.21 ± 1.41	0.088 ^ab^ **0.023 ^cd^** **0.020 ^ac^** 0.176 ^bd^
Thickness (µm)	118.70 ± 10.70	119.33 ± 13.92	156.67 ± 29.66	166.67 ± 10.99	0.914 ^ab^ 0.460 ^cd^ **0.024 ^ac^** **< 0.001 ^bd^**
Tensile strength (MPa)	14.20 ± 2.08	9.60 ± 0.55	6.82 ± 0.57	5.31 ± 0.62	0.054 ^ab^ 0.063 ^cd^ **0.022 ^ac^** **0.001 ^bd^**
Elongation (%)	2.51 ± 0.36	2.44 ± 0.36	3.13 ± 0.22	2.21 ± 0.38	0.823 ^ab^ **0.032 ^cd^** 0.076 ^ac^ 0.489 ^bd^
Elastic modulus (MPa)	927.12 ± 110.02	353.21 ± 20.21	172.49 ± 25.49	257.97 ± 31.80	0.111 ^ab^ 0.177 ^cd^ 0.079 ^ac^ 0.149 ^bd^
Transparency (%)	94.89 ± 0.45	63.78 ± 2.56	82.38 ± 0.98	69.92 ± 1.56	0.050 ^ab^ **0.033 ^cd^** **0.037 ^ac^** 0.203 ^bd^
L* (lightness)	25.99 ± 0.47	62.35 ± 1.65	46.67 ± 1.41	56.85 ± 0.44	**0.019^ab^** 0.066 ^cd^ **0.027^ac^** 0.167 ^bd^
a* (redness)	−1.43 ± 0.07	0.04 ± 0.39	0.39 ± 0.05	2.69 ± 1.01	0.158 ^ab^ 0.263 ^cd^ **0.003 ^ac^** 0.199 ^bd^
b* (yellowness)	10.74 ± 1.95	37.70 ± 1.51	26.49 ± 1.70	42.73 ± 2.31	**0.010 ^ab^** **0.036 ^cd^** **0.027 ^ac^** 0.230 ^bd^

(Results expressed as mean ± SD) a, b, c and d correspond to each type of film; Significant *p*-values are highlighted in bold.

**Table 2:** Barrier properties of the films: water vapor permeability.

Properties	Films				*p*-Values
	Polystyrene Petri Dishes		Lotus Negative Template		
	Without EO ^a^	With EO ^b^	Without EO ^c^	With EO ^d^	
WVTR (g/(m^2^.day))	31.53 ± 3.38	30.33 ± 3.04	27.23 ± 0.68	27.23 ± 4.73	0.672 ^ab^ 0.991 ^cd^ 0.156 ^ac^ 0.402 ^bd^
WVP (g/(Pa.day.m)) (×10^−6^)	2.74 ± 0.167	2.74 ± 0.274	3.58 ± 0.049	4.46 ± 0.178	1.000 ^ab^ **0.009 ^cd^** **0.009 ^ac^** **0.002 ^bd^**

(Results expressed as mean ± SD) a, b, c and d correspond to each type of film; Significant *p*-values are highlighted in bold.

**Table 3:** Contact angle, water solubility and weight loss of the films.

Properties	Films								*p*-Values
	Polystyrene Petri Dishes				Lotus Negative Template				
	Without EO ^A^		With EO ^B^		Without EO ^C^		With EO ^D^		
	Lower Side ^a^	Upper Side	Lower Side ^b^	Upper Side	Lower Side (Lotus Replica) ^c^	Upper Side ^d^	Lower Side (Lotus Replica) ^e^	Upper Side ^f^	
Contact angle (°)	48.27 ± 3.76	31.49 ± 1.05	71.80 ± 8.60	69.92 ± 3.02	81.95 ± 8.74	65.15 ± 3.11	112.50 ± 3.48	58.04 ± 5.71	**0.009 ^ab^** **< 0.001 ^ce^** **< 0.001 ^ac^** **0.001 ^be^** **0.001 ^cd^** **< 0.001 ^ef^**
Water solubility (%)	20.33 ± 1.45		23.41 ± 2.18		22.38 ± 0.73		22.38 ± 1.57		0.118 ^AB^ 0.546 ^CD^ 0.122 ^AC^ 1.000 ^BD^
Weight loss (%)	61.97 ± 3.16		56.61 ± 1.18		63.72 ± 2.82		47.66 ± 3.31		0.181 ^AB^ **0.003 ^CD^** 0.955 ^AC^ **0.031 ^BD^**

(Results expressed as mean ± SD) a, b, c, d, e and f correspond to each type of film (and side); A, B, C and D correspond to the films with or without the EO; Significant *p*-values are highlighted in bold.

**Table 4:** Antioxidant activity (β-carotene bleaching assay) and diameters of inhibition zones obtained with the films.

**Properties**	**Films**				***p*** **-values**
	**Polystyrene Petri Dishes**		**Lotus Negative Template**		
	**Without EO ^a^**	**With EO ^b^**	**Without EO ^c^**	**With EO ^d^**	
% Inhibition (β-carotene bleaching test)	3.45 ± 0.26	29.00 ± 1.67	5.91 ± 0.64	22.36 ± 1.29	**<0.001^ab^** **<0.001^cd^** **0.011^ac^** **0.007^bd^**
**Diameters of inhibition zones (mm)**	**Without EO ^a^**	**With EO ^b^**	**Without EO ^c^**	**With EO ^d^**	***p*** **-values**
*E. faecalis* ATCC 29219	6.45 ± 0.24	7.68 ± 0.39	6.52 ± 0.36	7.74 ± 0.40	**0.015 ^ab^** **0.018 ^cd^** 0.795 ^ac^ 0.861 ^bd^
*L. monocytogenes* LMG 16779	6.58 ± 0.33	8.25 ± 0.42	6.64 ± 0.35	8.46 ± 0.39	**0.007 ^ab^** **0.004 ^cd^** 0.840 ^ac^ 0.560 ^bd^

(Results expressed as mean ± SD) a, b, c and d correspond to each type of film; Significant *p*-values are highlighted in bold.
